# Comparison of knowledge, attitude, socioeconomic burden, and mental health disorders of COVID-19 pandemic between general population and health care workers in Egypt

**DOI:** 10.1186/s41983-021-00280-w

**Published:** 2021-02-15

**Authors:** Gellan K. Ahmed, Haidi Karam-Allah Ramadan, Samah Mohammed Refay, Mohamed A. Khashbah

**Affiliations:** 1 Department of Neurology and Psychiatry, Faculty of Medicine, Assiut University, Assiut, Egypt; 2 Department of Child & Adolescent Psychiatry, Institute of Psychiatry, Psychology & Neuroscience, King’s College London, London, SE5 8AF UK; 3 Department of Tropical Medicine and Gastroenterology, Faculty of Medicine, Assiut University, Assiut, Egypt; 4 Department of Clinical Pathology, South Egypt Cancer Institute, Assiut University, Assiut, Egypt; 5 Department of Economics, Neuroeconomics and Finance, Claremont Graduate University, Claremont, CA 91711 USA

**Keywords:** COVID-19, Anxiety, Depression, Obsessive compulsive, Mental health, Egypt

## Abstract

**Introduction:**

The global devastating effect of COVID-19 has caused anxiety and fear to variable extent among the public. We aimed to evaluate the knowledge, attitude, socioeconomic burden, and the mental health problems regarding anxiety, depression, and obsessive-compulsive disorder during COVID-19 on the general population and HCWs in Egypt.

**Methods:**

This study was conducted using a semi-structured online questionnaire in May 2020. Data on demographic features, socioeconomic scale, knowledge, and attitude regarding COVID-19 and the effect on different aspects of life were collected. Assessment was done using Arabic versions of *Beck’s* Anxiety Inventory, *Beck’s Depression Inventory–II*, and Yale-Brown Obsessive-Compulsive Scale. We divided participants into non-health care workers (non-HCWs) and HCWs groups.

**Results:**

There were 524 participants who responded to the survey from 23 governorates. More than half of the participants were females (57.4%), middle age (53%), and middle socioeconomic class (66.6%). Non-HCWs were 402 and HCWs were 122. Most participants had good knowledge about the disease and a positive attitude toward protective measures particularly in HCWs. COVID-19 showed negative impact on different aspects of participants’ life. HCWs had higher frequency of anxiety (32%) and OCD (29%) than non-HCWs (30% and 28%, respectively) while non-HCWs had higher depression (69%) than HCWs (66.4%). HCWs had higher rates of severe depression (20.5%) with moderate and severe OCD (4.9%, 1.6% respectively) than non-HCWs. Female gender, young age, urban residence, students, smoking, history of medical illness, and low socioeconomic class were significant associated factors.

**Conclusions:**

Health care workers had good knowledge about COVID-19 and a positive attitude toward the protective measures relative to non-HCWs. COVID-19 had a negative impact on different aspects of life and had a major association with the anxiety, depression, and OCD in both groups. Health professionals are more likely to have these psychological consequences.

**Supplementary Information:**

The online version contains supplementary material available at 10.1186/s41983-021-00280-w.

## Introduction

The coronavirus (SARS-CoV-2) that emerged in 2019 exerted the most devastating effects worldwide. It caused anxiety and fear among the public with the emergence and rapid spread of the disease. Such outcomes affected individuals at a global scale in varying degrees [1]. Similar to the SARS outbreak in 2003, knowledge and attitude toward infectious diseases are associated with various levels of panic, which can subsequently impact the prevention of the spread of the disease [2, 3].

Individuals due to COVID-19 suffer from stress, anxiety, and depression [4]. Moreover, the increased requirements for personal protective measures, such as the use of detergents, frequent handwashing, and cleaning of frequently used surfaces, could exert an impact on the symptoms of obsessive–compulsive disorder (OCD) in terms of incomplete cleanliness.

Cases of COVID-19 in Egypt were initially announced by the end of February 2020. The Egyptian government has made considerable efforts to allocate the required human and financial resources to contain the outbreak which continues to this day. Healthcare workers (HCWs) expressed concerns regarding the increased use of masks and sanitizers, which resulted in the shortage of resources in the medical market [5]. This concern was raised in addition to the shortage of healthcare facilities and personal protective equipment, thus endangering their health (WHO, 2020), and the stigmatization of HCWs in Egypt.

The different psychological impacts observed during the COVID-19 pandemic necessitate the evaluation of these domains among the Egyptian population. The limited resources of healthcare systems in Egypt, control measures for infection, and overall reduced level of public education could add to the psychological impact of the pandemic among the Egyptian population relative to other populations. Thus, the study hypothesized that the COVID-19 pandemic could exert a major effect on the mental health wellbeing of HCWs compared with the general population due to limited information. Furthermore, it could lead to increases in concerns about the disease without identifying treatments and in socioeconomic burden due to the lockdown strategies for reducing disease transmission. Against this background, this study evaluates the knowledge and attitude toward and socioeconomic burden of COVID-19 and assesses the associations of the COVID-19 pandemic with mental health problems regarding anxiety, depression, and obsessive–compulsive disorder of the general population and HCWs in Egypt.

## Methods

### Aim of the study

Therefore, we aimed to evaluate the knowledge, attitude, and socioeconomic burden of COVID-19 and to assess the associations of COVID-19 pandemic with the mental health problems regarding anxiety, depression, and obsessive-compulsive disorder of the general population and HCWs in Egypt.

### Study type

This was a cross-sectional study.

### Study design and population

The study employed a semi-structured questionnaire that was disseminated online using Google Forms between May 1 and June 1, 2020. The sample size was estimated using the EPI info statistical package Version 7. The parameters used to determine the sample size were a proportion of 0.5, a confidence level of 95%, and a margin of error of 5%. The sample size was 385 for the general population with 10% allocated as a non-response rate. A total of 524 participants responded. The study recruited the general population throughout Egypt. The participants were informed about the objective of the study and that the personal results were sent back through their e-mail (optional). The result report was sent at the end of survey with appropriate recommendation for need for psychiatric help in case of abnormal scoring of the questionnaires. They provided written informed consent before completing the questionnaire.

A link to the questionnaire was sent through WhatsApp and other social media platforms. The snowball sampling strategy was used. The participants were encouraged to forward the link to as many people as possible. After agreeing to participate, the participants filled up the questionnaire, which included a set of questions in sequential order over several sections as follows: demographic features, socioeconomic scale, knowledge and attitude toward COVID-19, concerns regarding COVID-19, and/or curfew and its effect on daily, social, and economic life. The last section of the questionnaire assessed mental health status using the Arabic versions of Beck’s Anxiety Inventory (BAI), Beck’s Depression Inventory-II (BDI-II), and the Yale–Brown Obsessive–Compulsive Scale (Y-BOCS). The participants were further grouped into two, namely, non-HCWs and HCWs. The study defined a HCW as a person who delivers medical care and services to the sick and ailing either directly as doctors and nurses or indirectly as aides, helpers, laboratory technicians, or even (workers in) medical waste [6].

The exclusion criteria were participants aged less than 18 years or who live outside Egypt.

### Ethical considerations

The study obtained ethical approval from the Institutional Review Board (IRB) of the Faculty of Medicine Assiut University with Approval Number 17300381. The study was registered on ClinicalTrials.gov with registration number NCT04344834 on April 14, 2020. Informed consent was written and obtained from the study participants by their agreement to participate in the study before filling the questionnaire. The participants were assured of data protection and informed that data would be anonymous. All procedures performed in this study were in accordance with the ethical standard of the institution and/or national research committee and with the 1964 Helsinki Declaration and its later amendments.

### Tools

#### Demographic features

The tool collected data on sex, age, residence, marital status, level of education, employment, number of children, smoking status, and history of chronic medical conditions.

#### Socioeconomic scale assessment scale for the family

The Arabic version of the socioeconomic scale consisted of four dimensions, namely, level of education, employment, total family monthly income, and lifestyle of the family [7].

#### Knowledge and attitude toward COVID-19

The tool included knowledge about routes of transmission, timeframe of developing the disease, protective measures used by the participants against COVID-19, expected percentage of recovery, frequency of following news about COVID-19, source and type of health information followed, and level of trust in health information.

Attitude toward COVID-19 covered dealing with self and other family members if a participant is a suspected case of COVID-19, dealing with suspected and recovered infected persons nearby, level of fear of the disease, expectation about the pandemic, and whether to accept voluntary isolation hospitals.

#### Social and economic burden of COVID-19 and/or curfew

Items under this dimension were used to assess the effect of COVID-19 and/or curfew on aspects of daily life, such as relationships with children and spouses and family problems. The effect of COVID-19 on daily activities, such as eating habits, sleep, spare time, travel, necessary activities, and family visits, and aspects of children’s life, such as physical, emotional, recreational, or educational, were assessed. Moreover, economic burden, such as a change in family income and work, was assessed.

#### The Beck’s anxiety inventory (BAI)

The study employed the Arabic version of the BAI [8], which is a multiple-choice self-reported inventory for measuring the severity of anxiety. The scale consists of 21 items. Each item is rated on a 4-point scale ranging from 0 (not at all) to 3 (severe). The total score ranges between 0 and 63, where the total scores of 0–7, between 8 and 15, between 16 and 25, and between 26 and 63 indicate normal, mild, moderate, and severe levels of anxiety, respectively [9].

#### The Beck’s Depression Inventory–II (BDI-II)

The scale is used to measure the severity of self-reported depression. The study utilized the Arabic version [10]. After summing the score of each item, the total score is obtained. The scale consists of 21 items with scores ranging from 0 to 63. Scores between 0 and 13, between 14 and 19, between 20 and 28, and between 29 and 63 indicate the absence of depression and mild, moderate, and severe levels of depression, respectively [11].

#### The Yale–Brown Obsessive–Compulsive Scale (Y-BOCS)

The Arabic version was used to specifically measure the types and severity of symptoms of OCD. The scale is composed of five items on obsessions and five items on compulsion with scores ranging from 0 to 4 (0 = no symptom, 1 = mild, 2 = moderate, 3 = severe, and 4 = extreme). The maximum total score is 40. The total scores of the range of severity for obsession and compulsion are as follows: 0–7 = subclinical (normal), 8–15 = mild, 16–23 = moderate, 24–31 = severe, and 32–40 = extreme. The cut-off score of clinically significant symptoms is 16 [12].

### Statistical analysis

Statistical analysis was performed out using SPSS version 21.0. Frequency distribution was used to summarize the categorical variables. Cross-tabulation was used to test for differences in categorical variables using the Chi-square test. An independent *t* test was used to compare continuous data. Univariable analyses using linear regression were conducted to identify the predictive factors of the occurrences of anxiety, depression, and OCD. Results were considered statistically significant at *p*-value ≤ 0.05.

## Results

### Demographic data and socioeconomic scale

A total of 524 participants responded from 23 governorates. Table 1 states that the participants comprised 402 non-HCWs and 122 HCWs (23.3%). More than half of the participants were female (57.4%), aged between 31 and 40 years (53.05%), married (58.8%), and belonged to the middle socioeconomic class (66.6%). University graduates accounted for approximately 42% of all participants. A history of chronic medical illnesses was observed in 18.3%, and approximately 83.2% were living in urban areas.

**Table 1:** Demographic features and socioeconomic scale of the included participants.

Item	Total (***n*** = 524) (***n***, %)	Non-HCWs (***n*** = 402) (***n***, %)	HCWs (***n*** = 122) (***n***, %)	***P*** value
**Sex:**				
Males	223 (42.6)	173 (43)	50 (41)	0.688
Females:	301 (57.4)	229 (57)	72 (59)	
**Age:**				
Less than 20 years	6 (1.14)	6 (1.5)	0 (0)	*P* < 0.000*
20–30 years	200 (38.2)	177 (44)	23 (18.9)	
31–40 years	278 (53.05)	186 (46.3)	92 (75.4)	
Older than 40 years	40 (7.6)	33 (8.2)	7 (5.7)	
**Distribution in Egypt:**				
Upper Egypt	440 (84)	71 (17.7)	13 (10.7)	0.065
Lower Egypt	84 (16)	331 (82.3)	109 (89.3)	
**Residence:**				
Rural	88 (16.8)	74 (18.4)	14 (11.5)	0.073
Urban	436 (83.2)	328 (81.6)	108 (88.5)	
**Marital status:**				
Single	196 (37.4)	171 (42.5)	25 (20.5)	*P* < 0.000*
Married	308 (58.8)	216 (53.7)	92 (75.4)	
Divorced	10 (1.9)	6 (1.5)	4 (3.3)	
Widow	10 (1.9)	9 (2.2)	1 (0.8)	
**Educational level:**				
Secondary school	32 (6.1)	32 (8)	0 (0)	*P* < 0.000*
University graduate	220 (42)	197 (49)	23 (18.9)	
University (Master)	174 (33.2)	124 (30.8)	50 (41)	
University (Doctorate)	98 (18.7)	49 (12.2)	49 (20.2)	
**Employment:**				
Employed	425(81.1)	303(75.4)	122(100)	*P* < 0.000*
Unemployed	72 (13.7)	72 (17.9)	0	
Retired	2 (0.4)	25 (6.2)	0	
Students	25 (4.8)	2 (0.5)	0	
**Number of children**				
No children	230 (43.9%)	201 (50%)	29 (23.8%)	*P* < 0.000*
Only one	72 (13.7)	57 (14.2)	15 (12.3)	
Two children	126 (24)	84 (20.9)	42 (34.4)	
> 2 children	96 (18.4)	60 (14.9)	36 (29.5)	
**Smoking:**				
Smokers	20 (3.8)	20 (5)	0 (0)	0.011*
Non-smokers	496 (94.7)	374 (93)	122 (100)	
Ex-smoker	8 (1.5)	8 (2)	0	
**History of chronic medical illness:**	96 (18.3)	69 (17.2)	27 (22.1)	0.214
**Socioeconomic class:**				
Low class	99 (18.9)	91 (22.6)	8 (6.6)	*P* < 0.000*
Middle class	349 (66.6)	270 (67.2)	79 (64.8)	
High class	76 (14.5)	41 (10.2)	35 (28.7)	

*HCWs* health care workers, *OCD* obsessive-compulsive disorder

*Significant *P* value

### Knowledge and attitude toward COVID-19

A significantly higher proportion of HCWs stated that transmission could occur through the nose, eyes, and stool relative to non-HCWs (i.e., 93.4% vs 85.1%, 69.7% vs 60.9%, and 24.6% vs 18.9%, respectively). Meanwhile, a higher percentage of non-HCWs cited transmission through the mouth (79.6%), contact with infected objects (52%), and food (19.9%). Moreover, more HCWs than non-HCWs believed that the disease develops within 14 days of exposure to a positive case (91.8%) and after contact with asymptomatic carriers (82.8%). In addition, approximately two-thirds of both groups expected recovery to be between 50 and 90%. More than two-thirds of the participants follow health information news on a daily basis with a higher proportion of HCWs (68%). A significantly high percentage reported that their sources of information are official records and social media. Significantly higher percent of HCWs follows the news regarding the dead cases (*p* = 0.001) (Table 2).

**Table 2:** Knowledge toward COVID-19 among the included participants

Variable	Non-HCWs (***n*** = 402) (***n***, %)	HCWs (***n*** = 122) (***n***, %)	***P*** value
**Routes of transmission of COVID-19:**			
Via the nose	342 (85.1%)	114 (93.4%)	0.016*
Via the mouth	320 (79.6%)	92 (75.4%)	0.323
Via the eyes	245 (60.9%)	85 (69.7%)	0.08
By contact with infected object	209 (52%)	59 (48.4%)	0.482
Through stool	76 (18.9%)	30 (24.6%)	0.171
By food	80 (19.9%)	16 (13.1%)	0.09
**Time period before developing the disease:**			
Within 14 days of exposure to a patient	350 (87.1%)	112 (91.8%)	0.156
After 14 days of exposure to a patient	90 (22.4%)	22 (18%)	0.304
After contact with asymptomatic carrier	295 (73.4%)	101 (82.8%)	0.034*
**Protective measures the participant use against COVID-19:**			
Frequent hand washing	372 (92.5%)	120 (98.4%)	0.019*
Reducing outdoor activities	352 (87.6%)	118 (96.7%)	0.004*
Cleaning objects you bring home	260 (64.7%)	80 (65.6%)	0.856
Wearing masks	184 (45.8%)	86(70.5%)	*P* < 0.000*
Using gloves	148 (36.8%)	74 (60.7%)	*P* < 0.000*
Using alcohol or detergents	240 (59.7%)	98 (80.3%)	*P* < 0.000*
Do not use protective measures	8 (2%)	0	0.116
**Expected percentage of recovery from the disease**^**a**^**:**			
Less than 10%	14 (3.5%)	0	0.932
Between 10 and 50%	60 (14.9%)	14 (11.5%)	
Between 50 and 90%	258 (64.2%)	74 (60.7%)	
More than 90%	70 (17.4%)	34 (27.9%)	
**Frequency of following the health information news**^**a**^**:**			
Daily	259 (64.4%)	83 (68%)	0.355
Weekly	8 (2%)	0	
Irregular	123 (30.6%)	37 (30.3%)	
Do not follow	18 (4.5%)	2 (1.6%)	
**The source of health information you use** ^**a**^**:**			
Official reports only	106 (26.4%)	38 (31.1%)	0.001*
Social media only	42 (10.4%)	0	
Both official records and social media	236 (58.7%)	82 (67.2%)	
**Type of the disease news they follow:**			
New infected cases	356 (88.6%)	110 (90.2%)	0.62
Died cases	199 (49.5%)	81 (66.4%)	0.001*
Recovered cases	187 (46.5%)	69 (56.6%)	0.052
**Level of trust of health information about COVID-19**^**a**^**:**			
Trust to somewhat	161 (40%)	55 (45.1%)	0.266
Sometimes trust	94 (23.4%)	30 (24.6%)	
Do not trust	95 (23.6%)	29 (23.8%)	
Always trust	52 (12.9%)	8(6.6%)	

*HCWs* health care workers

*Significant *P* value

^a^Question with only one answer

Table 3 presents the analysis of the attitude or concerns toward COVID-19. In terms of dealing with the self or a family member as a suspected case of COVID-19, 51.2% and 55.7% of non-HCWs and HCWs opted for home isolation. Moreover, 58.2% of the HCWs reported using protective measures in the presence of recovered COVID-19 cases, whereas a high proportion of non-HCWs (39.1%) would avoid any contact. Conversely, 71.6% of non-HCWs would use protective measure to deal with recovered cases and 8.5% would avoid any contact relative to the HCWs. Both groups significantly differed in level of fear from the disease (*p* = 0.038) with a higher percentage of HCWs reporting a moderate degree of fear.

**Table 3:** Attitude and concerns toward COVID-19 among the included participants in the study

Variable	Non-HCWs (***n*** = 402) (***n***, %)	HCWs (***n*** = 122) (***n***, %)	***P*** value
**How to deal if yourself or a family member is suspected to be infected with COVID-19:**			
Home isolation	206 (51.2%)	68 (55.7%)	0.384
Wait for symptoms to appear	175 (43.5%)	65 (53.3%)	0.058
Inform health authority	204 (50.7%)	56 (45.9%)	0.349
Go to hospital immediately	106 (26.4%)	34 (27.9%)	0.743
Consult friends or social media groups	68 (16.9%)	4 (3.3%)	*P* < 0.000^*^
**Dealing with suspected infected nearby person:**			
Contact with protective measures	195 (48.5%)	71 (58.2%)	0.061
Give advice to seek a doctor	178 (44.3%)	58 (47.5%)	0.526
Inform health authority	153(38.1%)	49 (40.2%)	0.676
Inform the surrounding to him	151 (37.6%)	37 (30.3%)	0.145
Avoid contact at all	157 (39.1%)	31 (25.4%)	0.006^*^
**Dealing with recovered nearby person:**			
Contact with protective measures	288 (71.6%)	82 (67.2%)	0.347
Contact in a normal way	158 (39.3%)	66 (54.1%)	0.004^*^
Inform surrounding to him	48 (11.9%)	8 (6.6%)	0.0.92
Avoid contact with him	34 (8.5%)	0	0.001
**Level of your fear from the infection**^**a**^**:**			
Very afraid	109 (27.1%)	23 (18.9%)	
Afraid to moderate degree	154 (38.3%)	54 (44.3%)	0.038^*^
Afraid to little degree	94 (23.4%)	38 (31.1%)	
Not afraid	45 (11.2%)	7 (5.7%)	
**Your expectation toward the epidemic**^**a**^**:**			
Better outcome	168 (41.8%)	44 (36.1%)	0.2
I do not know	155 (38.5%)	45 (36.9%)	
Worse outcome	79 (19.7%)	33 (27%)	
**Accept to volunteer in isolation hospitals**^**a**^**:**			
Yes	141 (35.1%)	43 (35.2%)	0.972
No	261 (64.9%)	79 (64.8%)	

*HCWs* health care workers

*Significant *P* value

^a^Question with only one answer

### Social and economic burden of COVID-19 and/or curfew

Analysis of the effect of COVID-19 and/or curfew on daily living demonstrated a significantly impaired social life especially regarding children’s problems for both groups with a more prominent effect among HCWs (22.1%). In contrast, non-HCWs (20.4%) reported an improvement in social life. COVID-19 and/or curfew exerted a significant effect on daily living with a higher effect in HCWs than non-HCWs mainly on family visits, travels, and eating habits. A significantly high proportion of HCWs indicated a worse impact of COVID-19 and/or curfew on their children’s life in the recreational, educational, and physical aspects. A significant effect of COVID-19 was observed for economic life in both groups (*p* = 0.008) mainly in the form of decreased income. Approximately one-third for both groups showed a decrease in working hours, which could be related to the curfew (Table 4).

**Table 4:** Impact of COVID-19 and/or curfew on different aspects of life of the included participants

Variable	Non-HCWs (***n*** = 402) (***n***, %)	HCWs (***n*** = 122) (***n***, %)	***P*** value
**Effect of COVID-19 and/or curfew on participant’s social life:**			
Increase in children problems	43 (10.7%)	27(22.1%)	0.001*
Increase family problems	59 (14.7%)	11 (9%)	0.107
Impaired marital life	20 (5%)	5 (4.1%)	0.687
Improved social life	82 (20.4%)	22 (18%)	0.566
**Aspects of participant’s daily life affected by COVID-19 and/or curfew:**			
Family visits	238 (59.2%)	88(72.1%)	0.01*
Necessary activities (e.g., bank, paying bills)	255 (63.4%)	83 (68%)	0.352
Spare time	208 (51.7%)	66 (54.1%)	0.648
Travel change	147 (36.6%)	61 (50%)	0.008*
Sleep troubles	145 (36.1)	51 (41.8%)	0.868
Eating habits change	125 (31.1%)	43 (35.2%)	0.028*
**Type of children life affected by COVID-19 and/or the curfew:**			
Recreational	217 (54%)	91 (74.6%)	*P* < 0.000*
Educational	135 (33.6%)	75 (61.5%)	*P* < 0.000*
Emotional	112 (27.9%)	42 (34.4%)	0.163
Physical	26 (6.5%)	20 (16.4%)	0.001*
**Effect of COVID-19 and/or curfew on participant’s economic life**^**a**^**:**			
No effect	118 (29.4%)	38 (31.1%)	0.008*
Decrease income	140 (34.8%)	42 (34.4%)	
Increase payments	86 (21.4%)	38 (31.1%)	
Decrease payments	50 (12.4%)	4 (3.3%)	
Increase income	8 (2%)	0	
**Effect of COVID-19 and/or curfew on participant’s work**^**a**^**:**			
No effect	123 (30.6%)	23 (18.9%)	*P* < 0.000*
Decrease working hours	143 (35.6%)	43 (35.2%)	
Vacation from work	96 (23.9%)	24 (19.7%)	
Fired from work	6 (1.5%)	0	
Increased risk during work	34 (8.5%)	32 (26.2%)	

*HCWs* health care workers

*Significant *P* value

^a^question with only one answer

### Analysis of Beck Anxiety Inventory, Beck Depression Inventory, and Yale-Brown Obsessive–Compulsive Scale

Table 5 indicates that HCWS experienced increased frequencies of anxiety (32%) and OCD (29%) than non-HCWs (30% and 28%, respectively), whereas non-HCWs had increased frequencies of depression (69%) than HCWs (66.4%).

**Table 5:** Frequency of severity scales of anxiety, depression, and OCD in the studied participants

	BAI score				BDI score				Y-BOCS			
	Total	Non-HCWs	HCWs	***P*** value	Total	Non-HCWs	HCWs	***P*** value	Total	Non-HCWs	HCWs	***P*** value
**Normal**	364 (69.5)	281 (69.9)	83 (68)	0.740	166 (31.7)	125 (31.1)	41 (33.6)	0.830	376 (71.8)	289 (71.9)	87 (71.3)	0.047*
**Mild**	42 (8)	30 (7.5)	12 (9.8)		156 (29.8)	120 (29.9)	36 (29.5)		128 (24.4)	101 (25.1)	27 (22.1)	
**Moderate**	62 (11.8)	46 (11.4)	16 (13.1)		100 (19.1)	80 (19.9)	20 (16.4)		18 (3.4)	12 (3)	6 (4.9)	
**Severe**	56 (10.6)	45 (11.2)	11 (9)		102 (19.4)	77 (19.2)	25 (20.5)		2 (0.4)	0	2 (1.6)	

*HCWs* health care workers, *BAI* Beck’s Anxiety Inventory, *BDI* Beck’s Depression Inventory–II, *Y-BOCS* Yale-Brown Obsessive-Compulsive Scale

*Significant *P* value

Analysis of the BAI scale demonstrated normal scores of 69.9% and 68% for HCWs and non-HCWs, respectively. Moderate degree of anxiety was higher in HCWs (13.1%), whereas those at a severe degree were higher in non-HCWs (11.2%). According to BDI scores, 31.1% and 33.6% of non-HCW and HCWs, respectively, indicated normal scores. Meanwhile, moderate degree of depression severity was higher in non-HCWs (19.9%), whereas those at a severe degree were higher in HCWs (20.5%). In addition, the Yale–Brown scale analysis illustrated that a normal score was the most frequent at 71.3% and 71.9% for HCWs and non-HCWs, respectively. Moderate and severe degrees of OCD reached 4.9% and 1.6% for HCWs and non-HCWs, respectively. No extreme score was recorded for both groups.

### Factors associated with anxiety, depression, and OCD in the participants

Regression analysis demonstrated that the most significant risk factors for anxiety were being less than 20 years old, urban residency, unemployed or student status, smoking, history of chronic diseases, and low socioeconomic class. Significant risk factors for depression were age being between 20 and 30 years old, student status, smokers, and low socioeconomic class. The observed significant risk factors for OCD were being female, urban residency, and history of chronic diseases (Supplementary Table 6).

## Discussion

Pandemics exert intense impacts on the mental health of a given population. Several studies reported that the general population adversely develops the psychological consequences of pandemics, such as SARS, Ebola, and H1N1 [13–16]. The common themes of psychological responses to outbreaks include guilt, grief from loss, stigmatization, anxiety, and depression. Fear and anxiety related to epidemics or pandemics also influence the behavior of communities. During the H1N1 epidemic, a significant proportion of the population was unaware of the severity and preventive measures of the epidemic [17].

To the best of our knowledge, the current study is the first to address the psychological association between COVID-19 and anxiety, depression, and OCD in Egyptian subjects from different governorates. More than half of the participants were female, middle-aged, married, and belong to the middle-class socioeconomic level. Majority of the participants were educated, urban residents, and employed. In a similar study on Egypt that assessed the knowledge and attitude of the general public toward COVID-19, two-third of the participants were female, most of them were from urban areas, and more than half were university graduates [18]. These characteristics are expected to be dominant because these population groups have more access to the Internet and thus complete the questionnaire.

Previous studies have found that the incubation period of SARS-CoV-2 could range between 0 and 24 days [19], and approximately 44% of the virus transmission can occur before symptoms emerge [20, 21]. Transmission occurs when the mouth, nose, or conjunctiva is exposed to infective droplets with certain evidence that COVID-19 could be present in fecal matter [22, 23]. The WHO (2020) has emphasized the importance of frequent hand washing, respiratory precaution, and environmental disinfection. Subsequently, the participants in the current study displayed an adequate level of knowledge regarding the mode of transmission of COVID-19, incubation period, and preventive measures. However, the HCWs utilized hand wash, masks, gloves, and alcohol more frequently than non-HCWs. Strict infection control measures in health services could increase the HCWs’ use of protective measures. Conversely, a shortage of such protective measures or high cost could be the reason for the low utilization of non-HCWs. Similarly, a study on the Egyptian population found that participants demonstrated sufficient general knowledge about COVID-19, its routes of transmission, and positive attitude toward preventive measures [18].

In the current study, the participants regularly followed health information mainly through official records and the Internet with a high level of interest in news about new positive and death cases related to COVID-19. The low level of interest in following recovered cases indicates concern regarding the spread and fatality of the disease particularly with the participants’ expectation of a low chance of recovery.

Attitude toward the management of suspected family members showed the tendency of the participants to avoid informing health authorities or seeking medical care in hospitals and preferring home isolation or waiting for symptoms to emerge. This tendency could be explained by the stigmatization of COVID-19-infected patients. The stigma can force people to hide their illnesses and avoid immediate healthcare (WHO, 2020). In a study on Egypt, approximately 22.7% of the participants believed that COVID-19 infection is associated with stigma and that 75% of the participants were willing to undergo home isolation, whereas a low proportion was willing to stay in hospitals in the case of contact with an infected case [18].

In the current study, the attitude of the non-HCWs toward positive or recovered cases nearby indicates a fear of transmission after recovery. However, a significant proportion of HCWs felt comfortable to contact the recovered cases safely. Nevertheless, Lo et al. [24] recently reported that the RNA of SARS-CoV-2 persists for approximately 18 days in the nasopharyngeal cavity or 19 days in the feces after the improvement of symptoms.

In the current study, the participants exhibited a moderate level of fear of the disease. More HCWs than non-HCWs expected worse outcomes from the epidemic. Moreover, COVID-19 exerted a negative impact on the different aspects of life of the participants, which added to the fear regarding the pandemic. This effect was more prominent in HCWs. This finding is similar to that of a study in Egypt that reported increased stress from work and home during the COVID-19 pandemic with increased financial stress [25]. In the current study, the non-HCWs reported more improvement in their social or daily life than HCWs, which could be attributed to the decrease in the working hours, thus leading to longer periods of staying at home due to the curfew and having more time for vacations. Meanwhile, the urgent increase in the demand for HCWs increased their working hours, thus putting more workload for them.

HCWs displayed increased frequencies in clinically significant symptoms of anxiety and OCD than non-HCWs. In addition, HCWS suffered from severe levels of depression and OCD than non-HCWs. Several studies provided evidence that COVID-19 is severely affecting the wellbeing of healthcare professionals [26]. This result could be related to the regular following of health information, increasing concern regarding news about infection and death rates, expectation of worse outcomes from the pandemic, negative impact on social and daily lives, decreased income, decreased vacation from work due to increased work shifts, and increased risk at work. In a systematic review, Vindegaard [27] demonstrated that symptoms of depression and anxiety increased among HCWs compared to the general public. Similarly, higher levels of symptoms of OCD were reported among health professionals compared with non-medical staff.

The current study illustrated that being female, young, students, smokers, and urban residents and a history of chronic diseases and low socioeconomic class were significant factors associated with high risks of anxiety, depression, and OCD. Similarly, a study in China reported that women and students suffered from high levels of anxiety and depression during the COVID-19 outbreak [1]. Similarly, previous study reported that those with chronic illnesses are susceptible to psychological impact as they consider themselves to be of poor health and thus more susceptible to contract COVID-19 [28].

In addition, urban residence increases the occurrence of these mental health outcomes because sources of health information, such as the Internet, are more readily available in urban areas. The mean score of knowledge could be significantly lower among those living in rural areas [18]. In Egypt, the diagnosis of COVID-19 cases occurs mainly in urban areas, where isolation facilities are present. Thus, residents in cities are more aware of such cases. Meanwhile, those belonging to the low socioeconomic class tend to suffer more from the impact of COVID-19 in the economic aspect. The COVID-19 pandemic led to several implications for closure of schools, companies, and public places, and changes in work routines that led to isolation and increased physical and social distance, and feelings of helplessness [29]. The dramatic economic impact [30] and lack of interpersonal attachments could lead to poor physical and mental health [31].

In another study in Egypt, Arafa et al. [32] demonstrated that being female, working in non-health sectors, watching or reading COVID-19 news for longer than 2 h daily, and lack of emotional support were associated with a high prevalence of severe to very severe depression and anxiety among the general population. However, the said study was conducted only in four Egyptian governorates.

Therefore, the present study recommends decreasing mental health consequences by encouraging daily exercise activities at home and maintaining safe modes of social communication, such as through smartphones, during the COVID-19 pandemic [33]. Early detection and effective treatment of mild clinical mood symptoms are necessary to prevent their evolution to more complex psychological responses [26]. Adequate psychiatric treatments should be provided for those presenting with severe mental health problems. Psychotherapy techniques based on the stress-adaptation model may be helpful because emotional and behavioral responses form part of an adaptive response to extreme stress [34, 35].

The present study has several limitations because it was limited to the individuals with access to smartphones, e-mail addresses, and the Internet. This group largely represents the educated population. Therefore, the findings should not be generalized across the population, particularly those with less educational attainment. Moreover, people with concerns regarding their mental health wellbeing were expected to participate as an online questionnaire was used to collect data.

## Conclusion

HCWs displayed sufficient knowledge about COVID-19 and held a positive attitude toward protective measures compared with non-HCWs. COVID-19 exerted a negative impact on the different aspects of daily, social, and economic life. HCWs displayed higher levels of anxiety and OCD than non-HCWs. Furthermore, HCWS suffered from high frequencies of clinically significant symptoms of depression and OCD in severe degrees than non-HCWs. Mental health surveillance among the public during or after the COVID-19 pandemic could promote adequate responses to the anticipated mental health issues associated with major public emergencies similar to COVID-19.

## Supplementary Information

**Additional file 1: Supplementary Table 6.** Risk factors associated with anxiety, depression, and obsessive-compulsive disorder in the studied participants

## Data Availability

All data generated or analyzed during this study are available from corresponding author on request.

## Abbreviations

COVID-19: Coronavirus-19
BAI: Beck Anxiety Inventory
BDI-II: Beck Depression Inventory
H1N1: Influenza A (H1N1) virus is the subtype of influenza A
SARS: Severe acute respiratory syndrome
RNA: Ribonucleic acid
Y-BOCS: Yale-Brown Obsessive–Compulsive Scale
OCD: Obsessive compulsive disorder
HCWs: Health care workers
