# Supplementary material for: Comparison of knowledge, attitude, socioeconomic burden, and mental health disorders of COVID-19 pandemic between general population and health care workers in Egypt
Source: Egypt J Neurol Psychiatr Neurosurg. 2021 Feb 15;57(1):25. doi: 10.1186/s41983-021-00280-w (PMC7883753; doi:10.1186/s41983-021-00280-w)
Supplement: Supplementary file 1 — Additional file 1: Supplementary Table 6. Risk factors associated with anxiety, depression, and obsessive-compulsive disorder in the studied participants [file 41983_2021_280_MOESM1_ESM.doc]

Supplementary Table 6: Risk factors associated with anxiety, depression and obsessive compulsive disorder in the studied participants.

| **Variable** | **Anxiety** | | | **Depression** | | | **OCD** | | |
| --- | --- | --- | --- | --- | --- | --- | --- | --- | --- |
|  | **R^2^** | **AR^2^** | **B(95%CI)** | **R^2^** | **AR^2^** | **B(95% CI)** | **R^2^** | **AR^2^** | **B(95%CI)** |
| **Gender** | 0.004 | 0.002 |  | 0.001 |  |  | 0.008 | 0.006 |  |
| Male |  |  | Reference |  | - .001 | Reference |  |  | Reference |
| Female |  |  | 1.463(-0.615 to 3.541) |  |  | 0.504(-1.244 to 2.252) |  |  | 1.162**(0.055 to 2.269) |
| **Age** | 0.013 | 0.007 |  | 0.022 | 0.016 |  | 0.011 | 0.005 |  |
| Less than 20 years |  |  | 7.875*(0.493 to 15.258) |  |  | 2.868(-3.306 to 9.042) |  |  | -3.412(-7.358 to 0.533) |
| 20 - 30 years |  |  | 1.449(-0.210 to 3.107) |  |  | 1.951**(0.564 to 3.339) |  |  | 0.391(-.0.496 to 1.278) |
| 31- 40 years |  |  | Reference |  |  | Reference |  |  | Reference |
| Older than 40 years |  |  | 1.159(-1.867 to 4.184) |  |  | -1.499(-4.029 to 1.032) |  |  | -0.929(-2.546 to 0.688) |
| **Residence** | 0.013 | 0.011 |  | 0.007 | 0.05 |  | 0.009 | 0.007 |  |
| Rural |  |  | Reference |  |  | Reference |  |  | Reference |
| Urban |  |  | 2.759*(0.672 to 4.846) |  |  | 1.757(-0.001 to 3.515) |  |  | 1.207*(0.091 to 2.324) |
| **Marital status** | 0.016 | -.010 |  | 0.011 | 0.005 |  | 0.011 | 0.005 |  |
| Married |  |  | Reference |  |  | Reference |  |  | Reference |
| Single |  |  | 0.07(-1.563 to 1.702) |  |  | 0.629(-0.746 to 2.003) |  |  | -0.810(-1.684 to 0.064) |
| Divorced |  |  | 4.868(-0.873 to 10.608) |  |  | 2.121(-2.713 to 6.955) |  |  | 1.587(-1.495 to 4.651) |
| Widow |  |  | -6.732*(-12.473 to -0.992) |  |  | -4.879*(-9.713 to -0.045 |  |  | -1.622(-4.695 to 1.451) |
| **Education level** | 0.034 | 0.027 |  | 0.023 | 0.015 |  | 0.006 | 0.001 |  |
| Secondary school |  |  | 2.824(-0.546 to 6.194) |  |  | 1.623(-1.218 to 4.463) |  |  | 1.451(-0.362 to 3.264) |
| University graduate |  |  | Reference |  |  | Reference |  |  | Reference |
| Master degree |  |  | -1.766(-3.573 to 0.041) |  |  | -1.288(-2.811 to 0.235) |  |  | 0.631(-0.341 to 1.604) |
| Doctorate degree |  |  | -2.282*(-4.445 to -0.119) |  |  | -1.801(-3.624 to 0.023) |  |  | 0.447(-0.717 to 1.611) |
| **Employment** | 0.006 | 0.004 |  | 0.003 | 0.001 |  | 0.001 | -.001 |  |
| Employed |  |  | Reference |  |  | Reference |  |  | Reference |
| Unemployed |  |  | 2.891*(0.557 to 5.226) |  |  | 1.954(-0.009 to 3.917) |  |  | 0.567(-0.688 to 1.822) |
| Retired |  |  | 1.488(-11.144 to 14.121) |  |  | 3.218(-7.405 to 13.841) |  |  | -4.017(-10.808 to 2.775) |
| Students |  |  | 5.288**(1.583 to 8.994) |  |  | 4.738**(1.622 to 7.854) |  |  | -1.475(-3.449 to 0.536) |
| HCWs |  |  | 1.529(-0.380 to 3.439) |  |  | 0.742(-0.863 to 2.348) |  |  | 0.467(-0.559 to 1.494) |
| **Working in isolation hospital** | 0.0001 | -.002 |  | 0.001 | -.001 |  | 0.0001 | -0.002 |  |
| Yes |  |  | -0.831(-5.699 to 4.038) |  |  | -1.355(-5.444 to 2.733) |  |  | 0.319(-2.281 to 2.918) |
| No |  |  | Reference |  |  | Reference |  |  | Reference |
| **Smoking status** | 0.018 | 0.014 |  | 0.013 | 0.009 |  | 0.006 | 0.002 |  |
| Smokers |  |  | 4.762*(0.696 to 8.828) |  |  | 3.846*(0.422 to 7.270) |  |  | Reference |
| Non-smokers |  |  | Reference |  |  | Reference |  |  | 0.856(-1.329 to 3.040) |
| Ex- smoker |  |  | 6.762*(0.408 to 13.116) |  |  | 3.996(-1.355 to 9.347) |  |  | 2.706(-0.708 to 6.119) |
| **History of chronic medical illness** | 0.03 | 0.028 | 4.104**(2.105 to 6.103) | 0.006 | 0.004 | 1.552(-0.148 to 3.251) | 0.01 | 0.008 | 1.232*(0.154 to 2.311) |
| Yes |  |  |  |  |  |  |  |  |  |
| No |  |  | Reference |  |  | Reference |  |  | Reference |
| **Socioeconomic class** | 0.018 | 0.014 |  | 0.019 | 0.016 |  | 0.006 | 0.002 |  |
| Low class |  |  | 2.773**(0.743 to 4.804) |  |  | 1.725*(0.02 to 3.429) |  |  | -0.782(-1.873 to 0.308) |
| Middle class |  |  | Reference |  |  | Reference |  |  | Reference |
| High class |  |  | -1.015(-3.272 to 1.243) |  |  | -2.006*(-3.900 to -0.112) |  |  | -0.773(-1.985 to 0.439) |

*p<0.05, **p<0.01
